# Intersigmoid Internal Hernia: Laparoscopic Repair of a Rare Cause of Bowel Obstruction

**DOI:** 10.1155/2022/5174496

**Published:** 2022-04-22

**Authors:** Ebrahim Almahmeed, Hasan Shawqi Aljawder, Mohamed Khalid Fadhul, Abdulmenem Abualsel

**Affiliations:** Department of General Surgery, King Hamad University Hospital, Bahrain

## Abstract

Intersigmoid hernia is a hernia of an abdominal viscus into the intersigmoid fossa, and it is one of the rare forms of internal hernia that can present with bowel obstruction. Intersigmoid hernia poses challenges in its diagnosis and treatment due to its rarity and vague signs and symptoms. As such, a high index of suspicion should be maintained, and early diagnostic examination should be done that includes an abdominal CT that may show signs suggestive of internal hernia. Managing this condition relies mainly on surgical intervention (open or laparoscopic) in a timely manner to prevent further complications, such as bowel ischemia. We present a case of intersigmoid hernia in a 51-year-old Bahraini male who presented with vague abdominal pain and vomiting.

## 1. Introduction

An internal abdominal hernia is defined as a protrusion of a viscus through a normal or abnormal mesenteric or peritoneal aperture [1]. In other terms, it is a rare cause of small-bowel obstruction with a percentage that does not exceed 5.8% [2]. A subtype of internal abdominal hernias is intersigmoid hernia, which is extremely rare and accounts for less than 6% of all internal hernias [3]. Despite the improvement in the investigational tests, it is still an under-diagnosed condition due to its vague symptoms and unspecified physical examination. CT abdomen is believed to facilitate the diagnosis of internal abdominal hernias [4]. However, surgical intervention is the cornerstone of management in these cases [5]. We present a case of intersigmoid internal hernia that was diagnosed in King Hamad University Hospital with abdominal CT and underwent laparoscopic surgical reduction and dissection of the hernia defect in a 51-year-old Bahraini male.

## 2. Case Presentation

We report a 51-year-old Bahraini retired male, who had a medical diagnosis of ischemic heart disease, with previous history of percutaneous coronary intervention and no past surgical history. The patient was complaining of intermittent left-sided abdominal and flank pain for a duration of 3 months. Additionally, it was associated with occasional vomiting of food content with history of alternating bowel habits.

The patient presented to the emergency department and was diagnosed clinically as left renal colic. He was investigated with non-contrast computed tomography (NCCT) in the urology clinic during his follow-up. The surgical team was notified by the radiology department of findings that a swirling sign in left lumbar region was present, raising the suspicion of internal hernia. The patient was called to the emergency department for further evaluation and management.

On clinical examination, his vital signs were within normal limits, with a heart rate of 94 beat/minute, BP 105/73 mmHg, temperature 36.9°C, RR 18 breath/minute, and oxygen saturation of 98% on room air. The patient was having mild left-sided abdominal pain. Additionally, his abdomen was soft and non-distended, with no visible superficial veins, and tenderness was noted on the epigastric and left upper quadrant region, with no guarding or rigidity, as well as no organomegaly. A digital rectal examination was performed, no palpable masses were felt, and there was no blood on the gloved finger.

The patient was further investigated with blood tests that were unremarkable and with no leukocytosis or acidosis [complete blood count, urea, electrolyte, serum creatinine, liver function tests, and venous blood gas].

**Figure 1:**
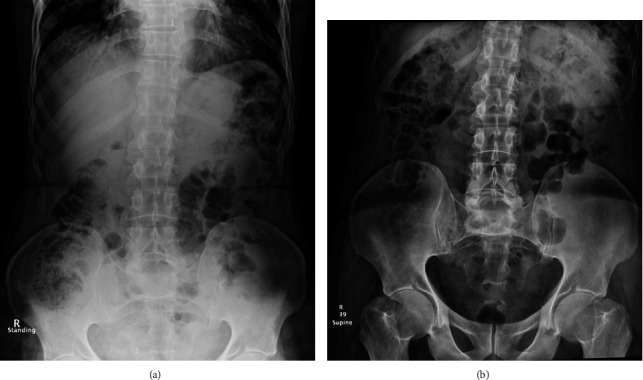
Erect (a) and supine (b) abdominal X-ray radiographs showing colonic fecal and gas content.

An erect chest X-ray was done and was unremarkable, with no subdiaphragmatic air, while an erect and supine abdominal X-rays showed only colonic fecal and gas content with no other abnormalities (Figure 1).

**Figure 2:**
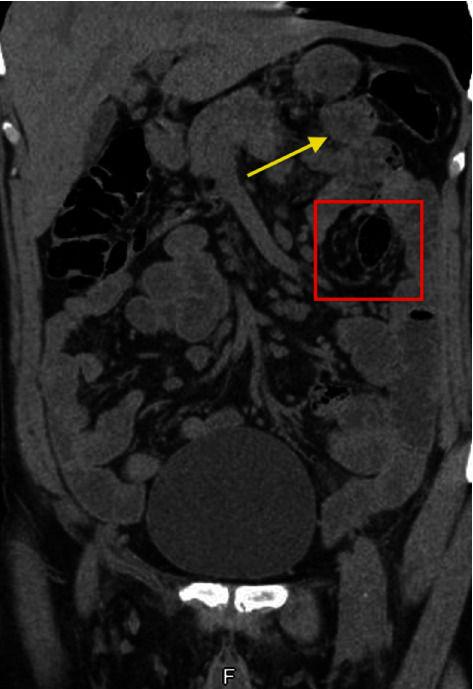
NCCT abdomen showing left lumbar regional mesenteric swirling sign (red box), and mildly dilated abnormally located proximal jejunal loops at the left upper quadrant (yellow arrow).

The previously done NCCT abdomen was reviewed and showed a left lumbar regional mesenteric swirling sign with mildly dilated abnormally located proximal jejunal loops at the left upper quadrant, more caudally projecting lateral to the medially located descending colon, raising the probability of internal hernia (Figure 2).

**Figure 3:**
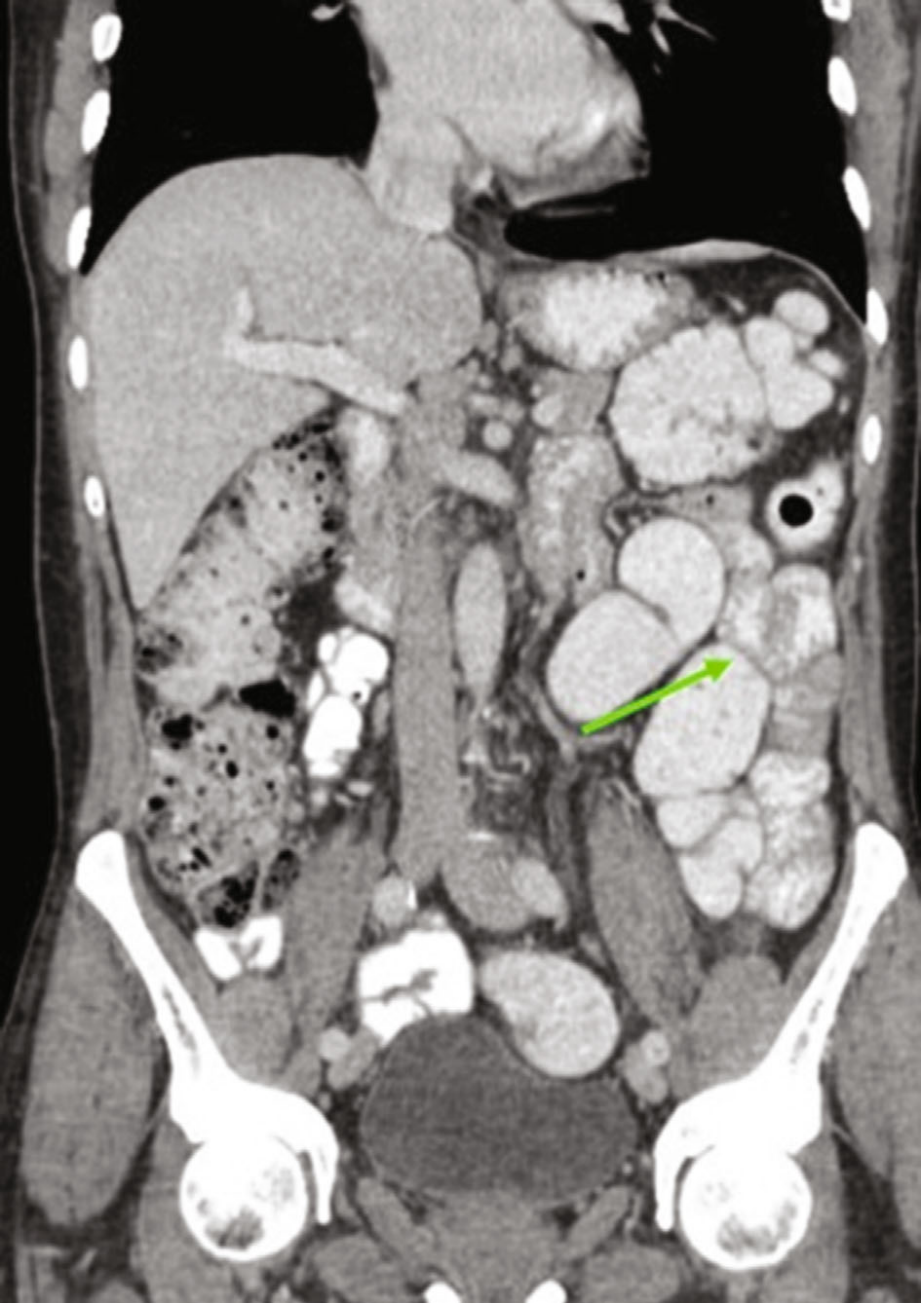
CT abdomen and pelvis with IV, oral, and rectal contrasts showing the jejunal loops project lateral to the sigmoid in the left lumbar region (green arrows).

The patient was admitted, kept nil per os, and was started on intravenous fluids. A CT abdomen and pelvis with IV, oral, and rectal contrasts was done, and showed regression of the previously seen left lumbar regional mesenteric swirling sign, with regression of the left upper quadrant located proximal jejunal loops distension, more caudally, the jejunal loops project lateral to the sigmoid in the left lumbar region suggesting medially located distal descending colon due to a dolichocolon extends up to left upper quadrant with no evidence bowel obstruction (Figure 3).

**Figure 4:**
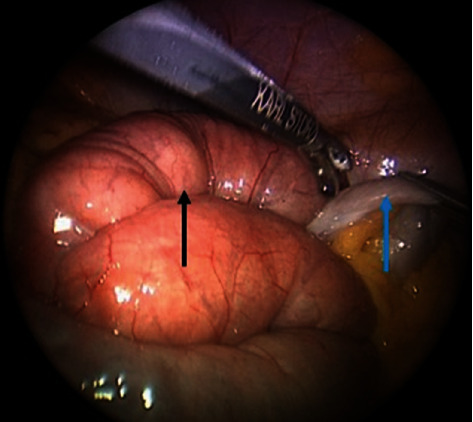
Diagnostic laparoscopy showing collapsed distal jejunal loops (blue arrow) and dilated proximal loops (black arrow).

**Figure 5:**
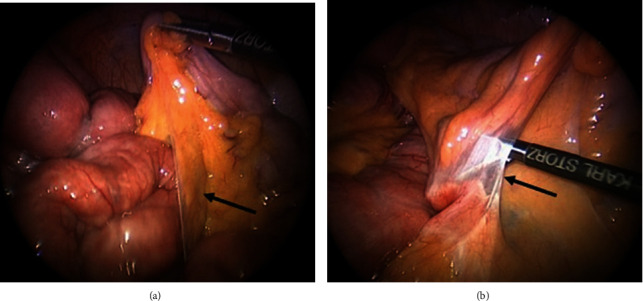
Diagnostic laparoscopy showing intersigmoid fossa (black arrow) containing jejunal loops.

The patient was taken to the operating room the following day where he underwent diagnostic laparoscopy which revealed collapsed distal jejunal loops and dilated proximal loops (Figure 4), a V-shaped intersigmoid fossa containing jejunal loops (Figure 5), and lateral abdominal wall adhesions creating a defect. The patient underwent uneventful laparoscopic reduction of internal hernia and dissection of intersigmoid fossa and adhesiolysis.

The patient was started on an oral diet on the same day, which he tolerated well. The following day, he passed motion and was vitally stable with no abdominal pain. He was discharged on day 1 postoperative with a follow-up appointment in the outpatient clinic, in which he was seen and discharged with no active complaints.

## 3. Discussion

Internal hernias which are defined as protrusion of an internal organ through an anatomical or pathological opening within the peritoneal cavity cause intestinal obstruction in up to 5.8% of all small-bowel obstructions [1]. They include paraduodenal, pericaecal, through the foramen of Winslow, transmesenteric, transomental, sigmoid mesocolon, and retroanastomotic hernias [2].

**Figure 6:**
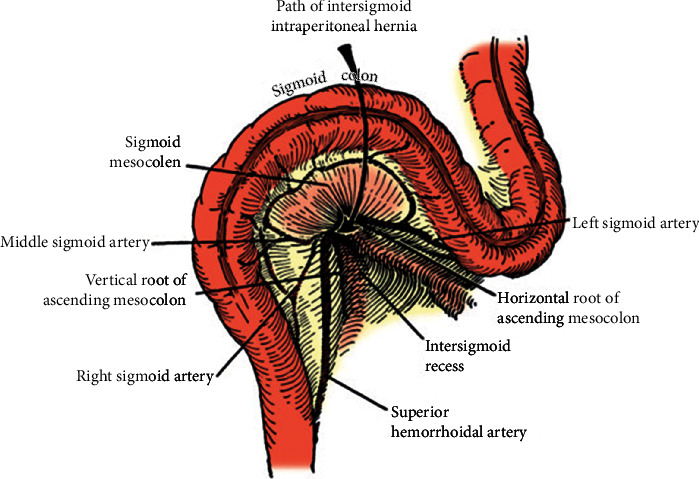
Intersigmoid hernia demonstration [7].

Sigmoid mesocolon internal hernias are further divided into intersigmoid, transmesosigmoid, and intramesosigmoid hernias which account for about 6% of internal hernias, and with intersigmoid hernia representing the majority of cases [3]. Intersigmoid hernia (Figure 6) occurs when herniated viscus protrudes through the intersigmoid fossa [4], which is a V-shaped peritoneal recess located at the level of the iliac crest, at the lateral aspect of sigmoid mesocolon with the left ureter lying underneath it [5, 6]. This fossa is congenital and is formed when there is a delay or incomplete fusion of the left periotoneal surface of the sigmoid mesentery with the parietal peritoneum of the posterior abdominal wall which normally occurs at 5 months of gestation [3, 5].

Our patient had an intersigmoid hernia as the hernia orifice represented by the intersigmoid fossa located on the fusion fascia in the dorsal sigmoid colon.

Clinically, patients with internal hernias can be asymptomatic with no distinctive findings upon history or physical examination. Moreover, they can present with significant symptoms which includes colicky abdominal pain, nausea, and vomiting as observed in our patient who was complaining mainly of colicky abdominal pain and frequent vomiting episodes. The severity of the symptoms is related to the presence or absence of incarcerated or strangulated viscus and to the duration of symptoms [8].

The best diagnostic non-invasive method for internal hernia especially in patients with evidence of obstruction is CT of the abdomen and pelvis which become the first-line imaging technique due to its availability, speed, and multiplanar reformatting capabilities [9]. CT findings might include a cluster of Y- and X-shaped dilated small-bowel loops entrapped behind the left posterior and lateral aspect of the sigmoid colon, with the defect located between sigmoid loops. In addition, findings of mesenteric vessel congestion can be seen [10]. If there are no signs of obstruction, barium enema can be performed, which can show sacculated ileal loops occupying the left lower quadrant with displacement of the sigmoid colon to the right [10].

Our case was diagnosed as intersigmoid hernia using CT of the abdomen which showed mesenteric swirling sign and dilated abnormally located proximal jejunal loops at the left upper quadrant.

The treatment approach for these patients is surgical exploration (laparotomy or laparoscopy) with reduction of the hernia and either obliteration or dissection of the intersigmoid fossa, and bowel resection might be necessary in cases of bowl ischemia [3].

Laparoscopic approach has the advantage of being both diagnostic and therapeutic, in addition to reduced postoperative pain, morbidity, and length of hospital stay when compared to laparotomy. On the other hand, laparoscopy is more challenging in cases of bowel distension and poses a risk for iatrogenic bowel injury [11].

Absolute contraindication to laparoscopic approach is related to contraindications to establish pneumoperitoneum such as hemodynamic instability or cardiopulmonary impairment [12].

Luckily our patient was diagnosed in a timely manner and surgical management using laparoscopic approach was successful.

There is no clear evidence in the literature regarding the recurrence rate of intersigmoid internal hernia.

## 4. Conclusion

Intersigmoid hernia is a rare cause of internal hernia which can be difficult to be diagnosed preoperatively due to the ambiguous signs and symptoms, all of which might lead to delay in the treatment. Prompt diagnosis is essential as delayed detection of the condition can lead to small-bowel ischemia and gangrene which increases the morbidity and mortality of patients. CT scan of the abdomen represents the main preoperative diagnostic method nowadays that allows precise localization of the abdominal obstruction. Surgical approach is the mainstay of management of intersigmoid hernia and should be performed as soon as possible.
